# Characterization of microRNA‐223‐3p as a novel promoter of cell proliferation and invasion in papillary thyroid carcinoma

**DOI:** 10.1002/ccs3.12057

**Published:** 2024-12-19

**Authors:** Xinghe Pan, Junliang Liu, Yitong Zhang, Chenglin Sun, You Li, Hongpeng Guo

**Affiliations:** ^1^ Department of General Surgery Central Hospital Affiliated to Shenyang Medical College Shenyang Liaoning China

**Keywords:** cell proliferation, epithelial‐mesenchymal transition, microRNA‐223‐3p, neurofibromatosis type 2, papillary thyroid carcinoma, tumor invasion

## Abstract

Papillary thyroid carcinoma (PTC), the most common thyroid cancer, has been linked to various molecular alterations. This study focuses on microRNA‐223‐3p, whose upregulated expression in PTC tissues appears to enhance tumor growth and cellular dysfunctions. Our findings demonstrate that microRNA‐223‐3p significantly promotes cell proliferation, invasion, and migration and induces epithelial‐mesenchymal transition (EMT). Additionally, neurofibromatosis type 2 (NF2) is identified as a direct target, suggesting that microRNA‐223‐3p could be crucial in PTC pathogenesis and may offer a target for therapeutic intervention.

## Graphical abstract

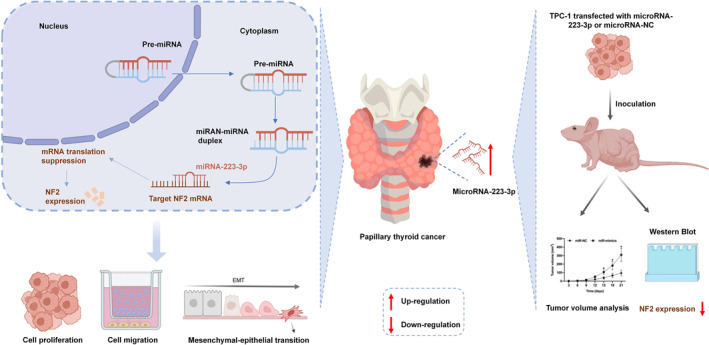

## 1 INTRODUCTION

Thyroid cancer (TC) is the most common endocrine malignant tumor globally, and its incidence has shown an increasing trend in recent decades.^1^, ^2^, ^3^, ^4^ In China, TC has become the fourth most common malignant tumor among women.^5^, ^6^, ^7^ Specifically, papillary thyroid carcinoma (PTC), as the most prevalent highly differentiated form, accounts for 85% of all TC cases.^8^, ^9^, ^10^ While PTC generally exhibits benign characteristics, the 5‐year survival rate of patients with metastatic PTC significantly drops to around 59% compared to approximately 97% for those without metastasis.^11^, ^12^, ^13^ This poor clinical prognosis underscores the importance of investigating the mechanisms of advanced PTC, identifying biomarkers, and developing new anticancer drugs to inhibit PTC progression.^14^

MicroRNA (miRNA) plays a key role in regulating gene expression and is a crucial molecule in the development of tumors.^15^ These small non‐coding RNA molecules inhibit protein expression by binding to the 3′ untranslated region of target genes.^16^ Aberrant miRNA expression is closely associated with the occurrence and progression of multiple cancers, including but not limited to lung cancer,^16^ liver cancer,^17^ breast cancer,^18^ and colorectal cancer.^19^ Notably, miRNA‐223, with its aberrant expression across different cancers, serves as a potential biomarker for many malignancies.^20^, ^21^, ^22^, ^23^

The regulatory role of miRNA‐223‐3p in various tumors has been well established, with extensive research on its functions in promoting or inhibiting proliferation, migration, and invasion of tumor cells.^24^, ^25^ For instance, miRNA‐223‐3p has been implicated in the biological processes of gastric cancer^22^ and renal cancer,^26^ among others, by targeting specific tumor‐suppressor genes or oncogenes. However, the expression of miRNA‐223‐3p in PTC and its relationship with patient prognosis have not been thoroughly investigated, nor have studies on its regulatory role in PTC progression been conducted.

Therefore, the objective of this study is to investigate the dysregulated expression of miRNA‐223‐3p in advanced PTC, assess its potential as a diagnostic biomarker for TC, and explore its regulatory role in PTC progression, including cell proliferation, cell cycle, migration, and invasion. By elucidating the molecular mechanisms by which miRNA‐223‐3p regulates PTC metastasis and establishing a xenograft model in nude mice to study its in vivo effects, this research study aims to provide crucial clinical insights for the early identification of PTC patients with lymph node or distant metastasis tendencies, as well as to identify new targets for the regulation of PTC metastasis and gene therapy. This study not only delves into the mechanisms of miRNA‐223‐3p in PTC but also offers new theoretical and experimental support for the future treatment and prognosis of PTC.

## 2 METHODS

### 2.1 Collection and preservation of samples

Between March 2023 and March 2024, our hospital collected samples from 68 patients diagnosed with PTC, along with adjacent thyroid papillary tissue samples. These specimens underwent no additional interventions prior to surgery and tissue sampling. Immediately after collection, the samples were preserved by freezing in liquid nitrogen to maintain their biological properties. Prior to surgery, all participating patients provided written informed consent, ensuring the ethical conduct of the research and respecting the patient’s autonomy. This study underwent review and approval by our institutional ethics committee to ensure compliance with ethical standards and regulations in all research activities.

### 2.2 Cell culture

The cell lines utilized in this study included TPC‐1, K1, and BCPAP, as well as the human thyroid follicular epithelial cells Nthy‐ori 3‐1 used as controls. These cell lines were purchased from the Shanghai branch of the American Type Culture Collection (ATCC) to ensure cell quality and reliability. During the cell culture process, the cells were placed in a constant temperature incubator at 37°C with 5% CO_2_. The culture medium used was DMEM (Dulbecco’s Modified Eagle Medium, Invitrogen, Carlsbad, CA, USA) supplemented with 10% fetal bovine serum (FBS, Thermo Fisher Scientific, Waltham, MA, USA) and 1% antibiotic solution (penicillin/streptomycin) to support cell growth and prevent bacterial contamination.

### 2.3 Quantitative real‐time PCR analysis method

In this study, we extracted total RNA from cultured cells and tissue samples of PTC patients using the TRIzol reagent (Thermo Fisher Scientific). The extracted RNA was assessed for concentration and purity using a NanoDrop spectrophotometer and agarose gel electrophoresis, followed by reverse transcription using the miRNA‐specific miScript II RT Kit (219600, Qiagen) according to the manufacturer’s protocol to synthesize cDNA.

To specifically quantify the expression of miR‐223‐3p, we used the miRNA Design V1.01 tool to design specific primers based on the mature hsa‐miR‐223‐3p sequence (UGUCAGUUUGUCAAAUACCCCA). The forward primer was GCGCGTGTCAAGTTTGCAAAT (Tm = 61.1°C, 21 nucleotides), and the reverse primer was AGTCAGGTCCGAGGTATT (Tm = 58.5°C, 20 nucleotides). We validated the specificity of these primers using the Oligo analysis tool to ensure no self‐complementarity or dimer formation risk. The optimized conditions for these primers ensured sensitivity and specificity in detecting the target miRNA.

For the qPCR reaction, we used the Taq PCR Master Mix Kit (Qiagen NV) and the above‐mentioned specific miRNA primers. The cDNA was diluted 10‐fold to meet experimental requirements for each reaction. The qPCR reaction conditions were as follows: pre‐denaturation at 95°C for 10 min, followed by 40 cycles consisting of denaturation at 95°C for 15 s and annealing/extension at the Tm temperature for 60 s. All qPCR experiments were performed on an ABI 7500 Real‐Time PCR System (Applied Biosystems, USA), using U6 as an internal reference gene to normalize miR‐223‐3p expression levels, with specific sequences listed in Table 1.

**TABLE 1:** RT‐qPCR primer sequences (human).

Name	Sequences (5′‐3′)
microRNA‐223‐3p (F)	GCGCGTGTCAGTTTGTCAAAT
microRNA‐223‐3p (R)	AGTGCAGGGTCCGAGGTATT
U6 (F)	TGGGGTTATACATTGTGAGAGGA
U6 (R)	GTGTGCTACGGAGTTCAGAGGTT

Using these optimized conditions and specific primers, we confirmed the expression level of miR‐223‐3p in PTC. The results demonstrated that the primers were efficient and reliable, consistent with previous experimental findings.

### 2.4 The overexpression of NF2 gene and transfection of MicroRNA‐223‐3p mimic in PTC TPC‐1 cells

In this study, to achieve overexpression of the NF2 gene, we utilized the NF2 overexpression lentivirus provided by Genepharm (Shanghai, China) and employed the GV358‐EGFP vector as a negative control. TPC‐1 cells were transfected by exposure to the lentiviral supernatant, followed by selection with 3 mg/ml of puromycin to obtain stable TPC‐1 cell lines overexpressing the NF2 gene (TPC‐1 NF2) and their corresponding negative control cell line (TPC‐1 NC). Furthermore, to investigate the role of microRNA‐223‐3p in PTC, we purchased the microRNA‐223‐3p mimic and its negative control (microRNA‐NC) from GenePharma (Shanghai, China) and performed transfection using Lipofectamine 2000 reagent (Invitrogen, USA). To more accurately simulate the in vivo expression environment, after 24 h of microRNA transfection, we further transfected the cells with NF2 overexpression plasmid.

### 2.5 Evaluation of cell proliferation activity using CCK‐8 assay

In this study, for an accurate assessment of cell proliferation ability, we utilized the CCK‐8 (Cell Counting Kit‐8) assay kit (Dojindo, Japan). Initially, the cells under investigation were evenly distributed into a 96‐well culture plate to monitor their proliferation status at different time points (1 day, 2 days, 3 days, 4 days, and 5 days). At each designated time point, CCK‐8 reagent was added to the respective wells, enabling us to indirectly evaluate cell viability by promoting a measurable color change through the interaction of water‐soluble tetrazolium salt with intracellular dehydrogenases. Subsequently, the absorbance values of each well in the plate were measured at a wavelength of 450 nm using a multimode reader automatic spectrophotometer produced by PerkinElmer (USA), where an increase in absorbance reflected an increase in cell quantity.

### 2.6 Cell cycle analysis using flow cytometry

In this study, flow cytometry was employed to analyze the cell cycle of cells from different treatment groups. Initially, post‐cultured cells were dispersed using a cell detachment solution (SIGMA, MO, USA) to ensure single‐cell acquisition. Subsequently, the cells were washed with phosphate‐buffered saline (PBS) to remove residues from the detachment solution. The cells were then fixed overnight in 70% ethanol for subsequent staining. Following fixation, the cells were washed again with PBS to remove ethanol and resuspended in a solution containing 100 μg/ml propidium iodide (Sigma) and 125 units/mL ribonuclease A (Sigma). Propidium iodide was utilized for DNA staining, while ribonuclease A was employed to digest RNA, thereby enhancing the specificity of DNA staining. Cell cycle analysis was performed using the FACScan flow cytometer (Becton Dickinson), and data was quantitatively analyzed using the CellQuest software package to ensure the accuracy and reliability of the results. Each sample collected a minimum of 10,000 cells to ensure statistical significance and accuracy.

### 2.7 Evaluation of invasive and migration capability of PTC cells using the transwell system

In this study, we utilized a 24‐well Transwell culture system to assess the invasive and migration ability of TPC‐1 cells. Initially, Matrigel (Corning, USA, product number 354230) was diluted and coated on the surface of the Transwell membrane to mimic the extracellular matrix and provide a three‐dimensional structural environment necessary for cell invasion. For migration, cells were processed without Matrigel. Subsequently, 0.2 ml of culture medium containing 5 × 10^4^ TPC‐1 cells was added to the upper chamber of the well, while 0.5 ml of serum‐containing medium was added to the lower chamber, with the serum acting as a chemotactic agent to attract cells to invade and migrate the lower chamber. After culturing for 2 days at 37°C and 5% CO_2_, cells that invaded and migrated the lower chamber were fixed with cold methanol on the underside of the membrane and stained using a 2% ethanol solution with 0.1% crystal violet for subsequent counting and analysis.

Following staining, cells were counted in 10 random fields of view at a 200× magnification to assess their invasive and migration capability. All images were captured using a Leica optical microscope from Wetzlar, Germany, ensuring image quality and accuracy of analysis.

### 2.8 Western Blot

In this study, an immunoblotting experiment (Western Blot) was utilized to detect the expression of specific proteins in breast PTC cells and nude mouse tumor tissues. Initially, total proteins were extracted from the samples using RIPA buffer (Sigma‐Aldrich), followed by protein concentration determination to ensure equal loading of 30 μg of protein lysates for SDS‐PAGE electrophoresis. After the protein electrophoresis was completed, the proteins were transferred onto a PVDF membrane for subsequent antibody recognition. The transferred PVDF membrane was incubated in PBS containing 5% skim milk to block nonspecific binding sites and reduce background signals. Following the incubation process, the membrane was separately incubated with the primary antibodies: E‐cadherin (14472S, 1:2000; CST), N‐cadherin (4061S, 1:1000; CST), Vimentin (5741S, 1:1000; CST), NF‐2 (12888, 1:1,000, CST), MMP2 (40994, 1:1000; CST), MMP9 (13667, 1:1000; CST). The selection of these antibodies was based on their crucial roles in tumor invasion and metastasis. Subsequently, the membranes were incubated with an anti‐rabbit IgG secondary antibody (A0208, 1:1000; Beyotime Institute of Biotechnology) for signal detection. For signal detection, an enhanced chemiluminescence (ECL) detection kit (Amersham) was employed to amplify the signal for visualization on radiographic films.

### 2.9 Exploring the regulatory role of microRNA‐223‐3p on NF‐2 expression using a dual‐luciferase reporter gene system

In this study, a dual‐luciferase reporter gene experiment was employed to investigate the regulatory effect of microRNA‐223‐3p on NF‐2 gene expression. Initially, the 3′UTR sequence of the NF‐2 gene was obtained through PCR, followed by its insertion into the pGL‐control vector (Promega) downstream of the Renilla luciferase gene to construct the reporter gene vector. To directly assess the impact of microRNA‐223‐3p on NF‐2 gene expression, mutations were introduced at the microRNA‐223‐3p binding site in the 3′UTR of NF‐2, creating a mutant reporter gene vector (pGL3‐NF‐2 MUT).

The 293T cell line was utilized in the experiment. Cells were transfected with Lipofectamine 2000 (Invitrogen) in combination with 20 nM microRNA‐223‐3p mimic or a control microRNA‐NC, along with the pGL3‐NF‐2 WT (wild‐type) or pGL3‐NF‐2 MUT (mutant) plasmids when they reached a density of 60%–80% growth, following the manufacturer’s instructions. After 48 h post‐transfection, the activity of Firefly luciferase and Renilla luciferase was measured using the dual‐luciferase reporter gene assay system (Promega). The experiment’s outcomes were evaluated by comparing the ratio of Firefly luciferase activity to Renilla luciferase activity, reflecting the regulatory effect of microRNA‐223‐3p on NF‐2 gene expression.

### 2.10 Establishment of nude mice model

A total of 20 female BALB/c nude mice were purchased from Liaoning Changsheng Biotechnology Co., Ltd. and housed in specialized facilities at the Experimental Animal Center of Shenyang Medical College to ensure consistency of experimental conditions and animal welfare. All experimental procedures were approved by the Institutional Animal Care and Use Committee of Shenyang Medical College (IACUC, Approval No. SYYXY2023010802) to ensure the ethical and regulatory compliance of the experiments.

In the experiment, TPC‐1 cells were transfected with microRNA‐223‐3p to mimic or control microRNA‐NC and then subcutaneously injected into the nude mice to establish a tumor growth model. At the endpoint of the experiment, when the tumors reached a certain stage of development, euthanasia was performed on the nude mice bearing TPC‐1 tumors using 200 mg/kg of pentobarbital sodium to minimize animal suffering. Subsequently, the tumors were excised for weighing to quantify the physical parameters of tumor growth.

To further analyze the biological characteristics of the tumor tissues, the immunohistochemistry (IHC) staining technique was employed to evaluate the expression of the Ki‐67 protein, a marker of cell proliferation that reflects the activity of tumor growth. Additionally, the expression level of NF‐2 protein in the tumor tissues was determined through immunoblotting method.

### 2.11 Statistical analysis methods

In this study, all data were based on results from at least three independent experiments and presented in the form of mean ± standard deviation (SD) to ensure the reliability and scientific validity of the outcomes. To assess the statistical significance between experimental data, we employed the independent samples *t*‐test to compare differences between two groups of data, and for comparisons involving more than two groups of data we utilized the analysis of variance (ANOVA) method. Both of these statistical analysis methods are effective in evaluating whether there are significant differences between the experimental groups.

Furthermore, to explore the relationship between the expression level of microRNA‐223‐3p and the clinical pathological characteristics of patients with PTC, we employed the chi‐square test. This method is suitable for analyzing associations in categorical data, revealing whether there is a statistical correlation between the level of microRNA‐223‐3p and the pathological characteristics.

## 3 RESULTS

### 3.1 Expression of microRNA‐223‐3p in PTC and its clinical stage correlation analysis

**FIGURE 1:**
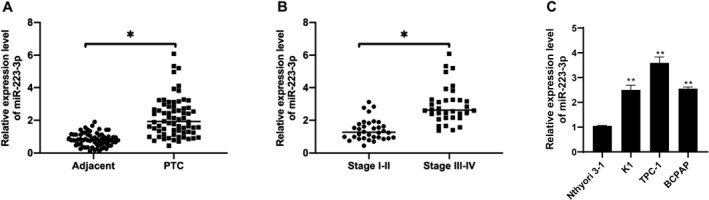
Expression analysis of microRNA‐223‐3p in PTC. (A) qRT‐PCR analysis results of the expression level of microRNA‐223‐3p in tumor tissues of 68 PTC patients compared to adjacent healthy thyroid tissues. (B) Comparison of the expression level of microRNA‐223‐3p in 68 PTC patients at different stages (I, II, and III, IV) using ANOVA for statistical analysis, where *p* < 0.05 indicates a significant difference. (C) Comparison of the expression level of microRNA‐223‐3p in PTC cell lines (TPC‐1, K1, and BCPAP) with normal thyroid follicular epithelial cells Nthyori 3‐1. Each sample was tested in three independent experiments, and the data are presented as mean ± standard deviation. Statistical significance was determined by unpaired Student’s *t*‐test, with *p* < 0.05 considered statistically significant.

To delve into the role and expression pattern of microRNA‐223‐3p in PTC, this study utilized qRT‐PCR to measure the levels of microRNA‐223‐3p in tumor tissues and adjacent healthy tissues of 68 PTC patients, as well as in several PTC cell lines (TPC‐1, K1, BCPAP) and human thyroid follicular epithelial cells Nthyori 3‐1. The research study revealed a significant increase in the expression level of microRNA‐223‐3p in PTC tumor tissues compared to that of the adjacent healthy tissues (Figure 1A), particularly in stages III and IV PTC patients, where the median expression of microRNA‐223‐3p was significantly higher than in stages I and II (Figure 1B). Furthermore, compared to Nthyori 3‐1 cells, PTC cell lines (including TPC‐1, K1, and BCPAP cells) exhibited a significant increase in the expression of microRNA‐223‐3p, with the highest levels observed in the TPC‐1 cell line (Figure 1C). Based on this observation, the TPC‐1 cell line was chosen as the primary model for subsequent functional experiments.

In conclusion, the expression level of microRNA‐223‐3p was significantly higher in the tumor tissues of PTC patients and PTC‐related cell lines compared to that of adjacent healthy tissues and normal thyroid cell lines. This finding suggests that microRNA‐223‐3p may play a crucial role in the development of PTC, particularly in the later stages of the disease. Therefore, the expression level of microRNA‐223‐3p may serve as a potential biomarker for the diagnosis and prognosis assessment of PTC.

### 3.2 The role of MicroRNA‐223‐3p in promoting PTC cell proliferation, invasion, migration, and EMT

**FIGURE 2:**
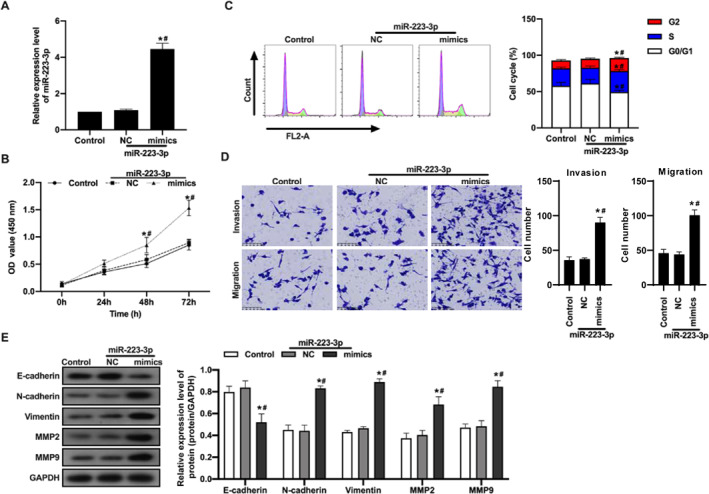
Functional impact evaluation of MicroRNA‐223‐3p in TPC‐1 cells. (A) Verify the transfection efficiency of microRNA‐223‐3p mimic by qRT‐PCR technology. (B) After transfection of cells with microRNA‐223‐3p mimic or NC, cell proliferation is evaluated using CCK‐8 reagent. (C) Analyze cell cycle distribution using flow cytometry after PI staining to assess the impact of microRNA‐223‐3p on the cell cycle of TPC‐1 cells. (D) Evaluate cell invasion and migration abilities using Transwell assay. Perform experiments using Transwell plates with Matrigel coating (invasion assay) and without Matrigel coating (migration assay). (E) Analyze the expression changes of EMT‐related proteins and MMPs by immunoblotting. Quantitative analysis of protein expression levels of E‐cadherin, N‐cadherin, Vimentin, MMP2, and MMP9 using specific antibodies. Data are presented as mean ± standard deviation of three independent experiments, and statistical differences are determined by unpaired Student’s *t*‐test or ANOVA. *p* < 0.05 is considered statistically significant. * indicates a significant difference compared to the control group, and # indicates a significant difference compared to the NC group.

To further investigate the role of microRNA‐223‐3p in regulating PTC cell behavior, this study transfected microRNA‐223‐3p mimic into TPC‐1 cells and comprehensively applied various biological techniques to evaluate its effects on TPC‐1 cells. Following transfection, qRT‐PCR was utilized to confirm the significant upregulation of microRNA‐223‐3p in TPC‐1 cells (Figure 2A), laying the foundation for subsequent functional analysis. Subsequent CCK‐8 proliferation assays indicated that treatment with the microRNA‐223‐3p mimics significantly enhanced the proliferation ability of TPC‐1 cells compared to that of the control group (NC) (Figure 2B). Flow cytometry analysis revealed an increase in the proportion of TPC‐1 cells in the G2 and S phases of the cell cycle after transfection with the microRNA‐223‐3p mimic, while the proportion of cells in the G0 phase significantly decreased (Figure 2C), indicating the regulatory role of microRNA‐223‐3p in the cell cycle progression. Furthermore, transwell assays were performed to assess cell invasion and migration capabilities, showing that TPC‐1 cells treated with the microRNA‐223‐3p mimic exhibited increased invasion and migration abilities (Figure 2D). Immunoblot analysis further confirmed that microRNA‐223‐3p promoted EMT by modulating the expression of related proteins, as evidenced by decreased expression of E‐cadherin and increased levels of N‐cadherin, Vimentin, MMP2, and MMP9 (Figure 2E).

In conclusion, the results of this study clearly demonstrate that upregulation of microRNA‐223‐3p in TPC‐1 cells significantly promotes cell proliferation, invasion, and migration abilities, while also enhancing EMT. These findings reveal the crucial regulatory role that microRNA‐223‐3p may play in the development and metastasis of PTC.

### 3.3 Investigating the role of microRNA‐223‐3p in targeting NF2 in PTC

**FIGURE 3:**
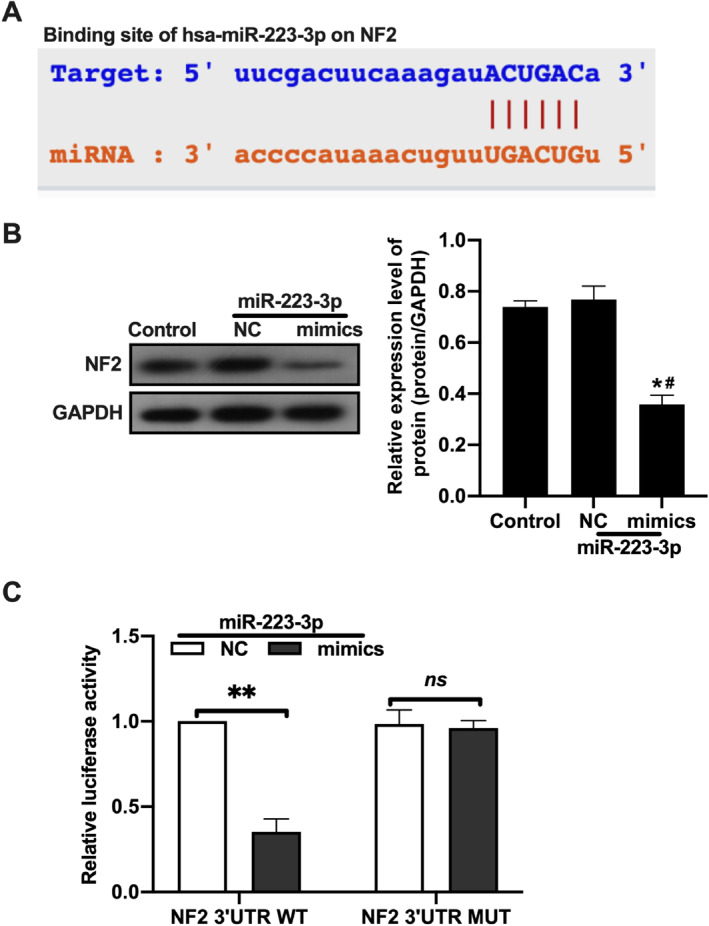
MicroRNA‐223‐3p regulates the expression of NF2 by targeting its 3′UTR. (A) Use bioinformatics tools to predict the specific binding sites of microRNA‐223‐3p in the 3′UTR region of NF2 mRNA. The prediction was carried out using the website www.targetscan.org, which illustrates the potential binding sequence of microRNA‐223‐3p with NF2 3′UTR. (B) Immunoblotting experiments were performed to detect the expression level of NF2 protein in TPC‐1 cells transfected with a microRNA‐223‐3p mimic. Specific antibodies against NF2 protein were used in the experiment to assess the impact of microRNA‐223‐3p on NF2 expression. (C) Validate the direct effect of microRNA‐223‐3p on NF2 3′UTR through a luciferase reporter gene assay. In the experiment, reporter gene plasmids containing wild‐type (WT) or mutant (MUT) sequences of NF2 3′UTR were co‐transfected with a microRNA‐223‐3p mimic or a control microRNA (NC). The influence of microRNA‐223‐3p on NF2 3′UTR was evaluated by measuring luciferase activity. Each experiment was repeated at least three times, with data presented as mean ± standard deviation. Statistical analysis was performed using unpaired Student’s *t*‐test or ANOVA, and *p* < 0.05 was considered statistically significant.

To delve into the molecular regulatory mechanisms of microRNA‐223‐3p in PTC cells, this study utilized bioinformatics methods to predict and identify potential target proteins of microRNA‐223‐3p. Using the website www.targetscan.org, we identified specific binding sites between the 3′UTR of NF2 and microRNA‐223‐3p (Figure 3A), suggesting that NF2 may be a direct target of microRNA‐223‐3p.

Further experimental validation demonstrated a significant downregulation of NF2 protein levels in the PTC cell model TPC‐1 following transfection with a microRNA‐223‐3p mimic, as observed through immunoblotting (Figure 3B), indicating that microRNA‐223‐3p regulates NF2 expression by directly targeting its 3′UTR. To validate this finding, a luciferase reporter gene experiment was designed, comparing the luciferase activity of co‐transfected cells with the microRNA‐223‐3p mimic and luciferase reporter genes containing either the wild‐type (WT) or mutant (MUT) sequences of NF2 3′UTR. The experimental results showed a significant decrease in luciferase activity of the reporter gene containing the NF2 3′UTR‐WT sequence under the influence of the microRNA‐223‐3p mimic compared to the microRNA‐negative control group, while the activity of the gene containing the MUT sequence was not significantly affected (Figure 3C), confirming NF2 as one of the specific target proteins of microRNA‐223‐3p.

In conclusion, this study not only predicted the direct interaction between microRNA‐223‐3p and NF2 through bioinformatics analysis but also validated, through in vitro experiments, the regulatory role of microRNA‐223‐3p in targeting NF2 expression by specific binding to its 3′UTR. This further elucidates the molecular mechanism through which microRNA‐223‐3p potentially plays a crucial regulatory role in PTC development via NF2.

### 3.4 MicroRNA‐223‐3p regulates TPC‐1 cell proliferation, invasion, migration, and EMT through targeting NF2

**FIGURE 4:**
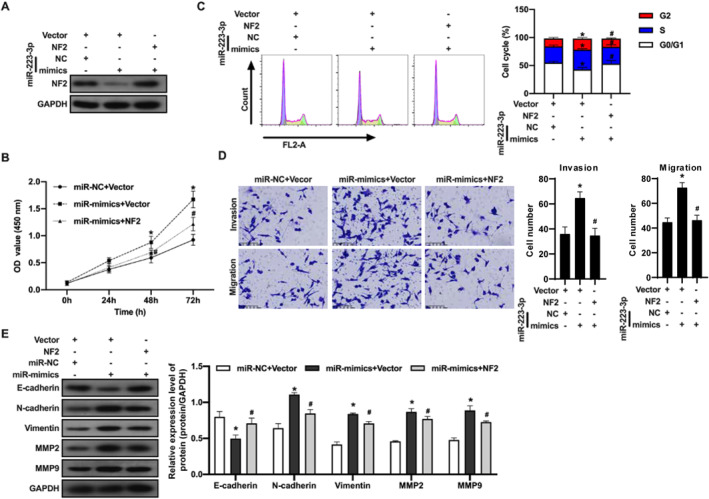
MicroRNA‐223‐3p mimic affects the biological behavior of TPC‐1 cells by targeting NF2. (A) The expression of NF2 protein after co‐transfection of microRNA‐223‐3p mimic and NF2 overexpression vector in TPC‐1 cells was detected by immunoblotting. Specific antibodies against NF2 were used to confirm transfection efficiency. (B) Cell proliferation ability was assessed using the CCK‐8 assay kit. Cells transfected with microRNA‐223‐3p mimic and/or NF2 overexpression vector were subjected to the CCK‐8 assay to measure cell proliferation activity. (C) Cell cycle distribution was analyzed by flow cytometry. After cell staining, flow cytometry was used to evaluate cell cycle changes in TPC‐1 cells after transfection. (D) Transwell assay evaluated cell invasion and migration ability. Cells were subjected to transfection, and then Transwell plates were used for invasion and migration assays to assess cell migration and invasion ability. (E) Immunoblotting analysis of EMT‐related proteins as well as MMP2 and MMP9 expression. After appropriate transfection treatment, protein samples were collected for immunoblotting analysis to evaluate changes in EMT‐related protein expression. The experiment was repeated three times, and data are presented as mean ± standard deviation. Statistical analysis was performed using unpaired Student’s *t*‐test or ANOVA, with *p* < 0.05 considered statistically significant. * indicates a significant difference compared to the microRNA‐NC + Vector group; # indicates a significant difference compared to the microRNA‐mimics + vector group.

To further elucidate the role of microRNA‐223‐3p in promoting PTC cell functions, especially its dependence on targeting NF2, this study conducted a series of in vitro experiments. By co‐transfecting microRNA‐223‐3p mimics and NF2‐overexpressing vectors into TPC‐1 cells, we validated the transfection efficiency using immunoblotting (Figure 4A). The experimental results revealed that the enhanced cell proliferation mediated by the microRNA‐223‐3p mimics was significantly inhibited under conditions of NF2 overexpression (Figure 4B).

Further cell cycle analysis demonstrated that NF2 overexpression could modulate the cell cycle alterations induced by the microRNA‐223‐3p mimics, specifically resulting in a decrease in the number of cells in the G2 and S phases and an increase in the number of cells in the G0/G1 phase (Figure 4C). Moreover, transwell invasion and migration assays showed that NF2 overexpression markedly attenuated the promoting effect of the microRNA‐223‐3p mimics on TPC‐1 cell invasion and migration abilities (Figure 4D).

The immunoblotting analysis further confirmed that NF2 overexpression effectively blocked the regulation of EMT‐related proteins MMP2 and MMP9 expression by the microRNA‐223‐3p mimics (Figure 4E). Collectively, these series of experimental results all point to a common conclusion: microRNA‐223‐3p exerts its function in promoting proliferation, invasion, migration, and EMT in TPC‐1 cells by targeting NF2.

### 3.5 MicroRNA‐223‐3p promotes TPC‐1 tumor cell proliferation In vivo by downregulating NF2

This study aimed to investigate the impact of microRNA‐223‐3p on the in vivo growth of PTC cell line TPC‐1, and further validate whether its mechanism of action involves the regulation targeting NF2 protein. By establishing a TPC‐1 cell xenograft tumor model in nude mice, we compared the tumor growth between the transfection of microRNA‐223‐3p mimic and control microRNA‐NC.

**FIGURE 5:**
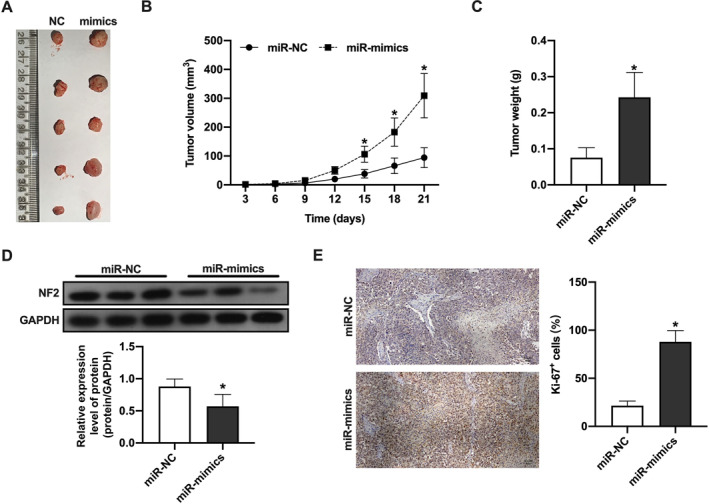
MicroRNA‐223‐3p Promotes Tumor Growth of TPC‐1 in Nude Mice. (A) Shows representative images of tumors from the transfection of the microRNA‐223‐3p mimic group and the control microRNA‐NC group. The tumor images were taken at the end of the experiment to visually display the differences in tumor volume. (B) The tumor volume growth curve was plotted based on data regularly measured during the nude mouse experiment, reflecting the comparison of tumor growth rates. (C) The weights of tumors in each group were statistically analyzed at the end of the experiment to quantify the effect of microRNA‐223‐3p on tumor growth. (D) The expression levels of NF2 protein in tumor tissues were detected by immunoblotting to validate the regulatory role of microRNA‐223‐3p on NF2 expression. (E) The number of Ki‐67 marked cells was analyzed through immunohistochemical staining to evaluate the cell proliferation activity in tumor tissues. The sample size for each group was *n* = 5. Statistical significance was determined using the Student’s *t*‐test, *p < 0.05 compared to the microRNA‐NC group, indicating a statistically significant difference.

The experimental results indicated that the tumor growth rate and average weight in the microRNA‐223‐3p mimic group significantly increased compared to the microRNA‐NC group, reflecting the role of microRNA‐223‐3p in promoting TPC‐1 cell proliferation and tumor growth (Figures 5A–C). Immunoblot analysis further revealed that the expression level of NF2 protein in the microRNA‐223‐3p mimic group was significantly decreased compared to the control group, suggesting that microRNA‐223‐3p may promote tumor growth by downregulating NF2 protein (Figure 5D). Additionally, immunohistochemical analysis of Ki‐67 expression showed that the cell proliferation induced by transfection with microRNA‐223‐3p mimic was significantly higher than that in the control group (Figure 5E), further confirming the crucial role of microRNA‐223‐3p in promoting TPC‐1 cell proliferation in nude mice.

In conclusion, our findings demonstrate that microRNA‐223‐3p significantly enhances the proliferation and tumor growth of TPC‐1 tumor cells in vivo, partly through its targeting and downregulation of NF2 protein.

## 4 DISCUSSION

PTC is the most common type of TC, accounting for approximately 80% of all cases, and its incidence has been steadily rising in multiple countries and regions.^8^, ^27^, ^28^, ^29^ While most PTC patients have a favorable prognosis, those with distant metastasis exhibit high recurrence rates and poorer outcomes.^30^, ^31^ Lymph node metastasis, a highly aggressive clinical‐pathological feature of PTC, is closely linked to local recurrence and distant metastasis of PTC.^28^, ^32^, ^33^, ^34^ Consequently, unraveling the intricate mechanisms of PTC metastasis is crucial for the early identification of aggressive PTC with metastatic potential and the discovery of effective treatment modalities. In contrast to prior studies, this research study not only delves into the expression and function of microRNA‐223‐3p in PTC but also elucidates its mechanism of action by targeting NF2, offering a new perspective on understanding the molecular underpinnings of PTC.

This study disclosed that the expression of microRNA‐223‐3p was significantly elevated in tumor tissues from 68 PTC patients as well as TPC‐1, K1, and BCPAP cell lines compared to adjacent normal tissues, and its aberrant expression correlated markedly with the staging of PTC patients. These results not only align with previous studies confirming elevated expression of microRNA‐223‐3p in renal cell carcinoma,^26^ colorectal cancer,^35^ prostate cancer,^36^ and lung cancer^37^ but further validate the promoting role of microRNA‐223‐3p in PTC development, particularly in facilitating PTC cell proliferation and increasing the number of cells in the S phase.

This study demonstrated that upregulation of microRNA‐223‐3p using a microRNA‐223‐3p mimic not only promotes PTC cell proliferation but also enhances cell migration and EMT while upregulating the expression of MMP‐2 and MMP‐9. Furthermore, our research study shows that miR‐223‐3p promotes PTC cell proliferation and invasion by downregulating NF2 expression, suggesting that NF2 may play a significant role in PTC. The NF2 gene encodes a tumor suppressor protein called Merlin, whose inactivation has been implicated in several cancers, such as neurofibromatosis, mesothelioma, breast cancer, and meningioma. In malignant mesothelioma, NF2/merlin inactivation is observed in approximately 40% of cases, indicating its critical role in cancer development and progression, which is associated with the activation of the Hippo and mTOR signaling pathways involved in cancer progression.^38^ In meningiomas, NF2 mutations are common, and NF2 loss is associated with increased cell proliferation and tumor growth. The merlin protein, as a negative regulator of mTORC1, when inactivated, leads to persistent mTORC1 activation, thereby promoting tumor growth.^39^ Additionally, NF2‐deficient meningiomas exhibits loss of contact‐dependent growth inhibition and enhanced cell proliferation, which is associated with increased expression and nuclear localization of Yes‐associated protein (YAP), suggesting that Merlin regulates cell growth by inhibiting YAP.^40^ Although reports of NF2 mutations in breast cancer are rare, the inactivation of NF2 in various tumor types, such as meningiomas and mesotheliomas, indicates that this gene plays an important role in tumorigenesis beyond the nervous system.^41^ Notably, in thyroid cancer, low NF2 expression is closely associated with tumor aggressiveness and poor prognosis in patients. These findings underscore the role of microRNA‐223‐3p as an oncogenic microRNA in PTC and reveal its mechanism of promoting PTC cell progression by directly targeting NF2, which contrasts with the role of NF2 in suppressing tumor progression, primarily by participating in cell adhesion and inhibiting EMT.^42^, ^43^, ^44^

By employing microRNA mimics and an overexpression system for NF2 protein, this study not only revealed the regulatory effects of microRNA‐223‐3p on PTC cell proliferation, cell cycle, migration, and EMT in vitro but also validated its promotion effects and NF2 downregulation in a nude mouse model. The application of these methods ensured the accuracy and reliability of the research results, and the consistency observed between in vivo and in vitro models further emphasizes the critical role of microRNA‐223‐3p in PTC development. While this study has made significant progress in elucidating the role and mechanism of microRNA‐223‐3p in PTC, there are some limitations, such as a relatively limited sample size, necessitating future studies for validation in larger patient cohorts. Additionally, this study primarily focused on the interaction between microRNA‐223‐3p and NF2; hence, future research studies could explore other potential targets of microRNA‐223‐3p in PTC and their underlying mechanisms.

In conclusion, this study confirms the elevated expression of microRNA‐223‐3p in PTC tissues, its correlation with tumor staging, and its role in promoting PTC cell proliferation, migration, invasion, and EMT through targeting NF2. It underscores the significance of microRNA‐223‐3p as a crucial oncogenic microRNA in PTC, providing a fresh perspective on understanding the molecular mechanisms in PTC and offering potential targets for targeted therapeutic strategies against microRNA‐223‐3p and NF2, showcasing their significant value in scientific and clinical domains. Future research studies will delve deeper into the role of microRNA‐223‐3p in PTC and other tumors and translate these molecular discoveries into effective therapeutic approaches for the treatment of PTC and other cancer types.

## 5 CONCLUSION

**FIGURE 6:**
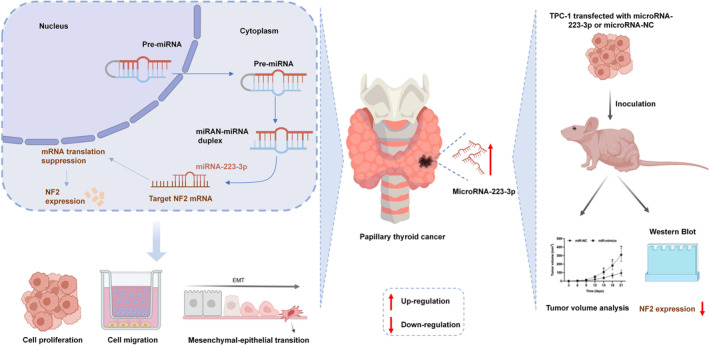
Exploring the role of MicroRNA‐223‐3p in the progression of PTC: a new mechanism targeting NF2.

Based on the aforementioned results, we can preliminarily draw the following conclusions: this study, for the first time, clearly identifies the overexpression of microRNA‐223‐3p in the field of PTC and, through a series of experiments, reveals its significant role in promoting PTC cell proliferation, migration, and EMT by targeting NF2 (Figure 6). This discovery not only provides a new perspective for understanding the molecular regulatory mechanisms of PTC but also offers potential molecular targets for targeted therapeutic strategies against PTC, holding significant scientific and clinical value.

## AUTHOR CONTRIBUTIONS

HPG, XHP contributed to the study concept and design. HPG, YL, JLL and YTZ contributed to the acquisition of data. HPG, JLL, CLS and XHP performed the statistical analysis. HPG, YL and XHP participated in interpretation of the data.

## CONFLICT OF INTEREST STATEMENT

The authors declare no conflicts of interest.

## ETHICS STATEMENT

All animal experiments were approved by IACUC of Shenyang Medical College (No. SYYXY2023010802). Clinical aspects of the study were overseen and approved by the ethics committee of Central Hospital Affiliated to Shenyang Medical College (NO. 2023039).

## Data Availability

All data can be provided as needed.
